# Mental Health and Human Rights of Women

**DOI:** 10.1192/j.eurpsy.2022.129

**Published:** 2022-09-01

**Authors:** M. Amering

**Affiliations:** Medical University of Vienna, Department Of Psychiatry And Psychotherapy, Vienna, Austria

**Keywords:** women’s mental health, women’s rights, Human Rights

## Abstract

Gender equality leads to better health and mental health for women and girls as well as to better public health and mental health for all. Inequality, discrimination and social exclusion are both cause and consequence of mental health problems for all and affecting women and girls in specific and substantial ways. Equality through the realization of non-discrimination, respect and enablement of autonomy as well as full inclusion in all spheres of life are demands of gender equality legislation as well as human rights obligations for persons with mental health problems. Essentials of non-discrimination laws concern key areas, including health, family planning, marriage and parenthood, employment, housing, education, standards of living and social, political and cultural participation, along with the right to be free from exploitation, violence and abuse. Gender-specific attention to the risks, rights and needs of women and girls and their families are legal obligations as well as clinical and scientific responsibilities. Because of the cumulative and interacting gender-based and other forms of discrimination, regulations such as those following the adoption of the UN-Convention on the Rights of Persons with Disabilities include specific provisions for women with psychosocial disabilities. Other examples for the urgent necessity of a gender-sensitive approach are – among many others - safety and gender-responsiveness of community and hospital settings, humanitarian crisis response, working with family carers, and of course, mental health teaching and research, including efforts towards gender parity in academic psychiatry.

**Disclosure:**

No significant relationships.

